# Anti-apoptotic effect of *Buchholzia coriacea* Engl. stem back extracts on AsPC-1 and mechanisms of action

**DOI:** 10.1186/s12906-021-03433-9

**Published:** 2021-10-09

**Authors:** Hope Onohuean, Rahmat Adetutu Adisa, Abdullateef Isiaka Alagbonsi

**Affiliations:** 1 Biopharmaceutics unit, Department of Pharmacology and Toxicology, Kampala International University Western Campus, Ishaka-Bushenyi, Uganda; 2 Department of Biochemistry, Faculty of Basic Medical Sciences, College of Medicine, University of Lagos, Idi-Araba, Lagos State Nigeria; 3 Department of Clinical Biology (Physiology Unit), School of Medicine and Pharmacy, University of Rwanda College of Medicine and Health Sciences, Huye, Rwanda

**Keywords:** Antioxidants, Apoptosis, AsPC-1 cell, *Buchholzia coriacea*, Caspase-3, Cell viability, Mitochondrial membrane potential, Free radical

## Abstract

**Ethnopharmacological relevance:**

*Buchholzia coriacea* Engl. is popularly called wonderful cola due to its wide ethnomedicinal use for the treatment of various ailments. We investigated the possible cytotoxic effect of its various fractions on human pancreatic cancer cell (AsPC-1) and also determined its mechanisms of action.

**Materials and methods:**

The AsPC-1 cells were cultivated and separately treated with 5-fluorouracil (5-FU) or *Buchholzia coriacea* Engl. bark (BC) (ethanol, aqueous, chloroform or ethyl acetate extract) for 72 h. Cell viability, caspase 3 and mitochondrial membrane potential (ΔΨm) were determined in vitro after the treatment. Nitric oxide (NO) and 2,2-diphenyl-1-picrylhydrazyl (DPPH) free radicals’ scavenging property, ferric reducing power and lipid peroxidation assays were also done to examine the antioxidant effect of BC in vitro*.*

**Results:**

Various extracts of BC, especially at 2500 μg/ml and 5000 μg/ml, increased the AsPC-1 viability while 5-FU decreased it. The activity of caspase 3 was increased by 5-FU but reduced by all concentrations of various extracts of BC. Incubation of AsPC-1 with 5-FU showed the majority of cells having the monomeric form of JC-1 dye (bright green fluorescence), which indicated de-energized mitochondria. However, fluorescence photomicrograph of cells incubated with different concentrations (20, 40 and 100 μg/ml) of BC extracts (aqueous, ethanol, chloroform and ethyl acetate) showed strong JC-1 aggregation (yellow), which indicated mitochondria with intact membrane potentials. BC extracts also scavenged NO and DPPH radicals, inhibited lipid peroxidation and increased ferric reduction, though not as much as ascorbic acid.

**Conclusion:**

This study suggests that BC elicits anti-apoptotic activity in AsPC-1 by increasing cell viability, decreasing caspase 3 activity, stabilizing the ∆Ψm, and scavenging free radicals. Even though BC is used ethnomedicinally as anti-cancer agent, our findings in the present study suggest that it has pro-cancer potential in-vitro, especially on pancreatic cells. Its anti-apoptotic activity in AsPC-1 could be of clinical significance, especially to counteract the effect of apoptotic agents on pancreatic cells.

## Introduction

Apoptosis, a programmed cell death, involves the process where cells die upon receiving certain stimuli [1] and understanding of its cellular mechanisms is key to the development of apoptotic genes’ target drugs. Apoptosis is important in physiological conditions, including involution of thymus in early age, shedding of the endometrium, regression of the lactating breast, the normal destruction of cells accompanied by replacement proliferation such as in the gut epithelium, and destruction in embryonic development for sculpting of tissue [2]. It is also important in pathological conditions such as anti-cancer drug-induced cell death in tumours, progressive cell death and depletion of cluster of differentiation 4 (CD4+) cells in Acquired Immunodeficiency Syndrome (AIDS), cell deaths due to injurious agents (like hypoxia, radiation and thermal injury), cell death in heart diseases like myocardial infarction, cytotoxic T cell-induced cell death, some forms of virus-induced cell death (e.g. Hepatitis B or C), etc.

Cancer results from an imbalance between cell division and cell death, and failure of cells meant to have died to receive a death signal. However, it has been inversely linked to the phenomenon of apoptosis. For instance, cancer may result from a succession of genetic changes during which healthy cell evades cell death, thereby transforming them into malicious ones often called malignancy [3]. Moreover, apoptosis eliminates potential malignant cells to curb hyperplasia and tumour progression, while downregulation of or resistance to apoptosis process leads to a full blow cancer or carcinogenesis [4]. Cells can evade apoptosis (and become malignant) when there is an imbalance between pro-apoptotic and anti-apoptotic proteins, caspase function downregulation and impaired signaling of death receptor [2].

Among the world’s deadliest malignant neoplasms is pancreatic cancer, which has been projected to be the second leading cause of cancer-related deaths by 2030 [5]. The incidence and mortality rate associated with pancreatic cancer have increased in many countries of the world, probably because of the low success rate in its treatment [6]. The conventional cancer treatment methods, including surgical resection, radiation therapy, and chemotherapy are not effective, expensive and less satisfactory; hence, curb of development of malignant cells and the proliferation of tumor cells are of importance.

Anti-cancer compounds or drugs elicit their mechanisms of action via promotion of apoptosis, which can be induced in cells through many mechanisms [7]. Compounds with potential ability to suppress the propagation of malignant cells by inducing apoptosis represent a valuable mechanistic method to both cancer chemoprevention and chemotherapy. The high cost, unfavourable side effects coupled with widespread and development of resistance to the presently-used anti-cancer agents [8] make it important to seek alternative ethnomedicinal therapy. Additionally, the use of bioactive, plant-derived component extracts as alternative cancer therapies has drawn the attention of researchers due to their low cost and less toxic effects [9].

Studies have shown that an array of plant-originated components have potentials of suppressing tumour development [10], and identifying such compounds may be an effective strategy for decreasing the incidence and severity of cancer., For instance, *Kielmeyera coriacea* (Clusiacaea) root, stem and leaf have been reported to have cytotoxic activity in tumour cells, such as human leukaemia (HL-60), human colon carcinoma (HCT-8), melanoma (MDA-MB-435) and glioblastoma (SF-295) [11, 12]. *Buchholzia coriacea* Engl. (BC, commonly called magic cola) seeds have been reported to have anti-microbial [13, 14], hypoglycaemic [15, 16], and anti-hypertensive effects [15]. Also, we have earlier reported its anti-inflammatory effect by enhancement of anti-oxidant defense system and possibly inhibition of release of inflammatory mediators [17]. Its bark has been traditionally used for the treatment of headache, sinusitis, otitis, pleurisy, bronchitis, ophthalmia, kidney pains and nasal congestion in Ivory Coast; to treat smallpox and skin-itch in Gabon and earache in Ghana [18].

In this study, the chloroform, ethanol, ethyl acetate, and aqueous extracts of the BC were evaluated for possible cytotoxic effect on pancreatic cancer cells (AsPC-1), and the involvement of free radicals in the effect was also investigated. Surprisingly, against our expectation, the BC extracts exhibited anti-apoptotic effect by increasing cell viability, decreasing caspase 3 activity, stabilising the ∆Ψm and scavenging free radicals in vitro*.*

## Methods

### Extraction and fractionation of BC

The BC was purchased from a farmer (who cultivated it) in Efon village of Ogun State, South-West of Nigeria, in compliance with the National Institute for Pharmaceutical Research and Development (NIPRD) and the Assessment of Herbal Medicines and Legislation by WHO. It was then authenticated by Miss O. O. Oyebanji in the Department of Botany, University of Lagos. The protocols for this study, including the permission for plant collection, were also approved by the Ethical Research Committee of the University of Lagos, Nigeria in addition to the authentication. A voucher specimen with a number LUH 5831 was deposited in the University Herbarium, University of Lagos, Lagos, Nigeria.

The BC was shade-dried and pulverised into powder; about 1600 g powder was placed onto two separate jars of 1000 g and 600 g respectively. They were defatted with n-hexane at room temperature by cold maceration with vigorous shaking for 72 h. The hexane extract was filtered out and the marc was allowed to dry. The marc stem bark was further re-extracted using 100% ethanol, filtered and concentrated in a rotary evaporator. To test for the biological activity, the dried crude ethanol extract was dissolved in dimethyl sulfoxide (DMSO) to a concentration of 100 mg/ml stock solution; this was later mixed with the culture media Dulbecco’s Modified Eagle Medium (DMEM) to achieve the desired concentration.

Ethanol crude extract (60 g) was dissolved in 100 ml of distilled water and properly mixed, then transferred to a separating funnel. About 1.5 L of Chloroform was added, shaken and left overnight. About 1.5 L of ethyl acetate was also used to fractionate by running 200 ml each time. The sample partitioned, having the chloroform or ethyl acetate partition down the separating funnel while the aqueous fraction remains at the top of the separation funnel. The chloroform and the ethyl acetate fractions were collected, concentrated and oven-dried at 50 °C, and stored until needed. The aqueous fraction which was left was dried in the oven at 50 °C for 1 day.

Using weighing balance (DHAUS Machine Adventurer Pro), 100 mg of each of the extract and fractions was weighed and dissolved in 30 μl of DMSO (Dimethylsulfoxide) in sample bottles. Then, 5 ml of PBS (Phosphate Buffer Saline) was added and mixed using Vortex 5 Machine to ensure proper mixing. It was filtered using filter paper and funnel into new sample bottles. The samples were further filtered using a membrane filter to sterilise them into an ethylenediaminetetraacetic acid (EDTA) container and were kept in the freezer overnight.

### Phytochemical analysis

Standard methods were used to qualify the presence of alkaloids, phenols, cardiac glycosides, terpenoids, steroids, flavonoids, saponin, tannins, and phlobatannin [19, 20]. The phenolic acid content was determined by using the Folin-Ciocalteu assay as previously described [21]. An aliquot (1 ml) of extracts or standard solution of Gallic acid (20, 40, 40, 60, 80 and 100 μg/ml) was added to a 25 ml volumetric flask, containing 9 ml of distilled water. A reagent blank was prepared using distilled water. One millilitre of Folin-Ciocalteu phenol reagent was added to the mixture and shaken. After 5 min, 10 ml of 7% Na_2_CO_3_ solution was added to the mixture. The volume was then made up to the mark. After incubation for 90 min at room temperature, the absorbance against the reagent blank was determined at 550 nm with a UV/Vis spectrophotometer. The phenolic acid content was expressed as mg Gallic acid equivalents per 100 g (mg GAE/100 g) of the extract.

Total flavonoid content was measured by the aluminum chloride colorimetric assay [21]. An aliquot (1 ml) of extracts or standard solutions of rutin was added to a 10 ml volumetric flask containing 4 ml of distilled water. To the flask, 0.30 ml of 5% NaNO_2_ was added and after 5 min, 0.3 ml of 10% AlCl_3_ was added. After 5 min, 2 ml of 1 M NaOH was added and the volume was made up to 10 ml with distilled water. The solution was mixed and absorbance was measured against the blank at 510 nm. The total flavonoids content was expressed as mg rutin equivalent per 100 g (mgRE/100 g) of dry extract.

### Cell lines and cultures

The AsPC-1 (Department of Cell Biology and Genetics, University of Lagos, Akoka) was cultured in RPMI medium (St. Louis, Mo, USA) supplemented with 10% (v/v) fetal bovine serum, 100 μg/ml penicillin, 20 μg/ml streptomycin (Sigma Aldrich Company, USA), 20 μg/ml kanamycin acid sulphate and 7.5% sodium carbonate solution. The cells were maintained as a monolayer in 25 cm^2^ plastic tissue culture flasks at 37 °C in a humidified atmosphere containing 5% CO_2_ in the air. Exponentially growing cells were harvested after brief trypsinisation and used in all the experiments.

### Toxicity assay (MTT assay)

The study of cell viability and proliferation is very important for evaluating cell population responses to external factors such as growth factors, antibiotics and anti-cancer cancer drugs. The sciencell™ MTT cell viability and proliferation assay allow simple, accurate and reliable counting of metabolically active cells, based on the conversion of pale yellow MTT 3-[(4,5-Dimethylthiazol-2-yl)-2,5-diphenyltetrazolium bromide] (Science Cell laboratories; USA) to purple formation crystals, which can be solubilised and spectrophotometrically quantified.

Cells were grown in RPMI 1640 medium at 37 °C under 5% CO_2_ in a humidified incubator in a T-25 culture flask. Cells were trypsinised, harvested, counted and seeded into a 96-well plate at a density of 1.8 × 10^6^ cells/ml and incubated for 24 h for the cells to attach to the plate before the addition of the test compounds. Then, 50 mg of each of the BC extracts was dissolved in 30 μl DMSO and made up to 5 ml with PBS. The extracts were filter-sterilised and applied in various concentrations (5000, 2500, 1250, 625, 312.50, 156.25, 78.13, 39.06, 19.53, 9.77 μg/ml). About one-tenth of the positive standard 5-FU (Tabade Pharmaceuticals Yaba, Lagos) were also added to some wells. Blank and control wells were included. The plate was then incubated for 72 h. The MTT solution was prepared (5 mg/ml in PBS), 10 μl of which was added to each of the 96 wells at the end of the 72 h incubation period. The plate was wrapped in aluminum foil and incubated at 37 °C for 3 h. Then, 100 μl of MTT solubilisation buffer was added to each well afterwards. The plate was then mixed gently by shaking, and the optical density (OD) was read in triplicate with a microplate reader at 492 nm and 630 nm (background absorbance). IC50 values were determined by plotting a linear regression curve. The per cent cell viability was calculated as follows:

$$\mathrm{Cell}\kern0.17em \mathrm{viability}\;\left(\%\right)=\frac{\mathrm{OD}\;\mathrm{of}\kern0.17em \mathrm{treatment}}{\mathrm{OD}\;\mathrm{of}\kern0.17em \mathrm{control}}\times 100$$

### Caspase-3 activity assay

Cells were grown in RPMI 1640 medium at 37 °C under 5% CO_2_ in a humidified incubator in a T-25 culture flask. Cells were trypsinised, harvested, counted and seeded into a 96-well plate at a density of 1.8 × 10^6^ cells/ml and incubated for 24 h for the cells to attach to the plate before the addition of the test compounds. After 24 h incubation, cells were treated with 20 and 5 μg/ml of each extract as well as 2 μg/ml of 5-FU for the positive standard, l0 μg/ml of 5-FU was added to the cells for the positive control (induced apoptosis). Then, 2.5 μl of Z-VAD-FMK inhibitor (final concentration of 50 μM) was added to the cells at the same time as 5-FU was added for the inhibited apoptosis samples. Negative control wells (containing untreated cells) were also prepared. The plate was incubated in a humidified environment at 37^0^c for 72 h. The medium was aspirated from each well; cells were rinsed with ice-cold PBS, trypsinised and harvested into properly-labelled Eppendorf tubes and centrifuged at 5000 rpm for 10 mins. The supernatant was discarded and the cell pellets were washed in ice-cold PBS and centrifuged again at 5000 rpm for 10 min after which the supernatant was discarded. The cell pellets were re-suspended in cell lysis buffer (50 μl) and were lysed by repeated freeze-thaw cycles to ensure complete lysis. The lysates were centrifuged at 15000 rpm for 20 min. The supernatant fraction (cell extract) was collected and was used to measure caspase activity following the kit manufacturer procedure.

### Mitochondrial membrane potential (Δψm) analysis

The Δψm was analysed using JC-1 (Promega Cayman Chemical Company, USA.). JC-1, a fluorescent compound, exists as a monomer at low concentrations and form aggregates at higher concentrations. Cells were exposed to extract for 24 h at various concentrations of 20μg/ml, 40μg/ml, and 100μg/ml. Control cells were grown in medium alone. Then, the cells were incubated in 0.5 ml of medium containing JC-1 (2.5 μg/ml) for 30 min at 37 °C, and images were taken using a Carl-Zeiss epifluorescence microscope with a triple band-pass filter at central research laboratory, University of Lagos Teaching Hospital, Lagos. The fluorescence of the JC-1 monomer was green, whereas that of the aggregate was orange-yellow. Mitochondria with intact membrane potentials concentrate JC-1 into aggregates; hence, the mitochondria fluoresce orange-yellow. De-energised (decreased ΔΨm) mitochondria cannot concentrate JC-1 and fluoresce green [22]. For quantitative estimation, Image J analyser software (NIH Image, Maryland, USA) was used to count the number of JC-1 monomers and aggregates and their ratio was calculated for each group.

### Nitric oxide scavenging activity assay

A 2 ml of 10 mM sodium nitroprusside dissolved in 0.5 ml phosphate buffer saline (PH 7.4) was mixed with 0.5 ml of BC extracts at various concentration (0.25, 0.50, 0.75, and 1.0 mg/ml). The mixture was incubated at room temperature for 150 min. Then, 1 ml of incubated solution was mixed with 1 ml of Nedd reagent. The mixture was incubated at room temperature for 30 min. Absorbance was measured using a spectrophotometer at 540 nm. Ascorbic acid was used as standard. Blank was 1 ml of water, 2 ml of sodium nitroprusside and 1 ml of Nedd reagent. Control was 2 ml of sodium nitroprusside, 0.5 ml of phosphate buffer, 1 ml of Nedd reagent and 0.5 ml of methanol. The number of nitric oxide radicals scavenged was determined using the formula shown below:

$$\%\mathrm{Inhibition}=\left[\left(\mathrm{Ac}\hbox{-} \mathrm{As}\right)/\mathrm{Ac}\;\right]\ast 100$$

Where Ac = Absorbance of the control, As = Absorbance of the plant extract.

### 2,2-diphenyl-1-picrylhydrazyl (DPPH) free radical scavenging activity assay

The DPPH radical scavenging activity was determined according to the procedure described by [23]. An aliquot of 2 ml of 0.04% DPPH solution in ethanol and 1.0 ml of BC extracts/garlic acid at various concentrations were mixed. The mixture was shaken vigorously and allowed to reach a steady-state at room temperature for 30 min in a dark chamber. Decolourisation of DPPH was determined by measuring the absorbance at 517 nm. A control was prepared using 1 ml ethanol mixed with 2 ml of DPPH. The percentage inhibition of DPPH radicals by the extract/compound was determined by comparing the absorbance values of the control and the experimental tubes. The percentage of inhibition was calculated using:

% Inhibition = [(Ac-As)/ Ac] * 100; where Ac is absorbance of control; As is the absorbance of extracts/ascorbic acid. IC50 value (a concentration at 50% inhibition) was determined from the curve between percentage inhibition and concentration. All determinations were done in duplicate and the IC50 value was calculated by using the equation of the line.

### Determination of ferric reducing power

The Fe^3+^-reducing powers of the four BC extracts were determined as previously reported with slight modification [24]. Different concentrations (25–100 μg/ml) of the extracts (1.0 ml) were mixed with 2.5 ml phosphate buffer (pH 7.0) and 2.5 ml potassium ferricyanide (1%), followed by incubation at 50 °C for 30 min. After incubation, 2.5 ml of trichloroacetic acid (TCA, 10%) was added to terminate the reaction and centrifuged at 3000 rpm for 10 min. The upper portion of the solution (2.5 ml) was mixed with 2.5 ml distilled water, and 0.5 ml FeCl_3_ solution (0.1%) was added. The reaction mixture was left for 10 min at room temperature and the absorbance was measured at 700 nm against an appropriate blank solution. Ascorbic acid was used as a positive control/standard and all tests were performed in duplicate. Higher absorbance of the reaction mixture indicated greater reducing power.

### Determination of lipid peroxidation

A quantity of 0.1 ml of the homogenate was pipetted into test tubes and 0.1 ml of 0.01 M Tris HCl Buffer was added, then 0.1 ml of 0.16 mM ferrous ammonia sulphate was also added and 0.1 ml of 0.06 mM Ascorbic acid was added, then the various concentrations of BC extracts were added to the test tubes and incubated for 1 h, then 0.2 ml of 8.1% sodium dodecyl sulphate was added. Then, 1.3 ml of 10% TCA and 1 ml of TBA was also added and boiled for 20 min. The control contained 0.1 ml of homogenate, 0.1 ml of 0.01 M Tris HCl buffer, 0.1 ml of 0.16 mM ferrous ammonia sulphate, 0.1 ml of 0.06 mM ascorbic acid and 0.1 ml of water. Absorbance was taken at 532 nm. Ascorbic acid was used for the standard.

### Statistical analysis

Statistical evaluations were done using statistical package for social sciences (SPSS) version 16.0. (IBM Corporation, Armonk, NY). All values given were the Mean ± S.E.M. of the variables measured. Comparisons among the groups were done by one-way analysis of variance (ANOVA), followed by *posthoc* Tukey multiple comparison test. Statistical significance was considered at *p* < 0.05.

## Results

### Qualitative and quantitative analysis of BC

The results revealed the presence of alkaloids, phenols, glycosides, terpenoids, flavonoids, saponins, tannins, and phlobatannins in aqueous and ethanol fractions while terpenoids are present in chloroform fraction. Phenols and Flavonoids are also present, while other phytochemicals are absent in chloroform and ethyl acetate fractions of BC (Table 1).

**Table 1:** Phytochemical analysis of extracts of *B. coriacea* stem bark

Phytochemicals	Aqueous Extract	Chloroform extract	Ethanol Extract	Ethylacetate extract
Alkaloids	+	–	+	–
Phenols	+	+	+	+
Cardiac glycosides	+	–	+	–
Terpenoids	+	+	+	–
Steriods	–	–	–	–
Flavonoids	+	+	+	+
Saponins	+	–	+	–
Tannins	+	–	+	–
Phlobatannins	+	–	+	–

+ denotes present, − denotes absent

The total flavonoids content of the four different extracts is higher than the total phenol content. Chloroform extract has the highest content of phenol and flavonoids (Table 2).

**Table 2:** Quantitative analysis of total phenols and flavonoids in extracts of *B. coriacea* stem bark

Extract fractions	Total phenol (mg GAE/100 g)	Total flavonoids (mg RE/100 g)
Aqueous	10.851	48.3
Chloroform	39.265	53.67
Ethanol	17.293	50.338
Ethylacetate	20.327	52.5

*GAE* denotes gallic acid equivalent, *RE* denotes rutin equivalent

### 5-FU decreased but extracts of BC increased AsPC-1 viability

**Fig. 1:**
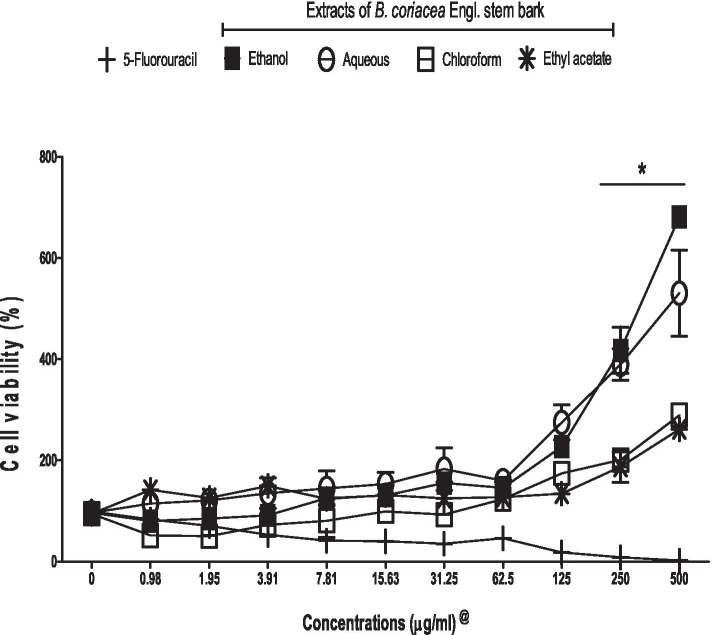
Effects of 5-fluorouracil and *B. coriacea* stem back extracts on AsPC-1 viability. *p < 0.05 vs control; ^@^denotes that the concentrations of 5-fluorouracil are × 10^− 1^ of their corresponding extracts

All concentrations of 5-FU significantly decreased AsPC-1 viability when compared to control. It was also observed that the percentage of cell viability was inversely proportional to the 5-FU concentration. However, various extracts of BC, especially at 2500 μg/ml and 5000 μg/ml, increased the AsPC-1 viability when compared to control (Fig. 1).

### Extracts of BC decreased caspase 3 activity in AsPC-1

**Fig. 2:**
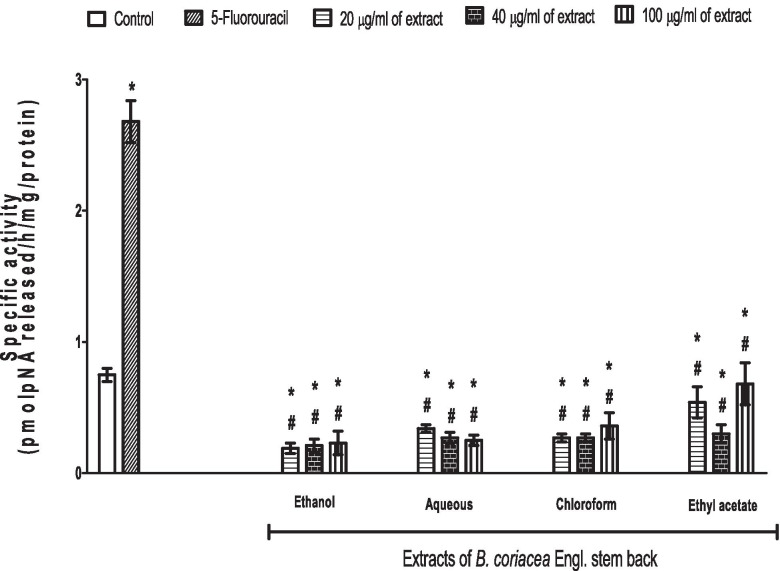
Effect of 5-fluorouracil and *B*. coriacea stem bark extracts on caspase-3 activity in AsPC-1 cell. *p < 0.05 vs. control; ^#^*p* < 0.05 vs. 5-fluorouracil

Caspase 3 is an executor caspase that serves as a link for the intrinsic and extrinsic pathways. The activity of caspase 3 was increased by 5-FU when compared to control, supporting the anti-cancer (pro-apoptotic) effect of 5-FU. However, the activity of caspase 3 was reduced by all concentrations of various extracts (ethanol, aqueous, chloroform and ethyl acetate) of BC when compared to control and 5-FU (Fig. 2).

### Extracts of BC stabilise the membrane

**Fig. 3:**
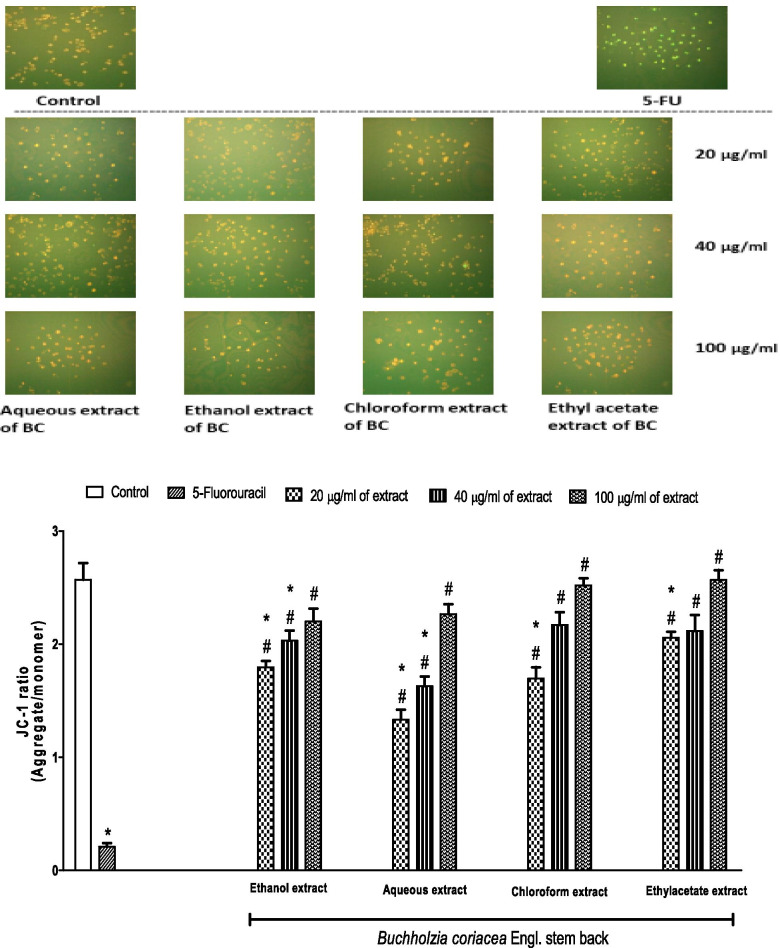
Effects of 5-fluorouracil and *B. coriacea* stem bark extracts (BC) on the membrane potential of AsPC-1 cell. *p < 0.05 vs. control; ^#^*p* < 0.05 vs. 5-fluorouracil; image resolution is 640 × 480

A cellular functional assay was performed to estimate the spatial variation in mitochondrial membrane potential (Δψm) using JC-1. This compound accumulates in the mitochondria as a result of the membrane potential. At low Δψm, mitochondria accumulate fewer JC-1 molecules and fluoresce green, whereas at high concentrations (high Δψm), the compound aggregates and exhibits a yellow fluorescence. Incubation of AsPC-1 cells with 5-FU showed a majority of cells having the monomeric form of JC-1 dye (bright green fluorescence), indicating de-energised mitochondria. However, fluorescence photomicrograph of cells incubated with various concentrations (20, 40 and 100 μg/ml) of different extracts of BC (aqueous, ethanol, chloroform and ethyl acetate) showed strong JC-1 aggregation (yellow), indicating mitochondria with intact membrane potentials as control. We further calculated the ratio of JC-1 aggregate: monomer for each group. Our data further showed that cells incubated with 5-FU had lower aggregate: monomer when compared with the control. However, various concentrations (20, 40 and 100 μg/ml) of different extracts of BC (aqueous, ethanol, chloroform and ethyl acetate) had higher aggregate: monomer when compared to those incubated with 5-FU. It was also noted that cells incubated with 100 μg/ml of each of the extracts and those incubated with 40 μg/ml of chloroform and ethyl acetate extracts did not show any significant difference from the control (Fig. 3).

### Extracts of BC scavenge NO radical in vitro

**Fig. 4:**
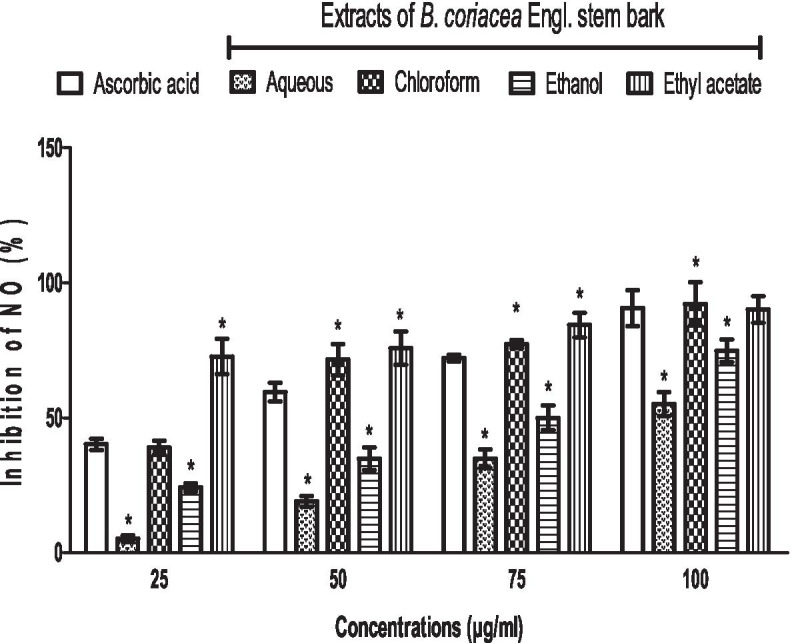
Scavenging activity of *B. coriacea* stem bark extracts on NO radicals in vitro. *p < 0.05 vs. ascorbic acid (control)

The inhibitions of nitric oxide (NO) by the control substance (vitamin C) and BC extracts were dose-dependent and increased as concentrations increased, which reached a peak at 100 μg/ml. Ethyl acetate extract of BC caused the highest (followed by chloroform extract), while aqueous extract of BC caused the lowest inhibition (followed by ethanol extract) of NO across all concentrations when compared to control (vitamin C) (Fig. 4).

### Extracts of BC scavenge DPPH radical in vitro

**Fig. 5:**
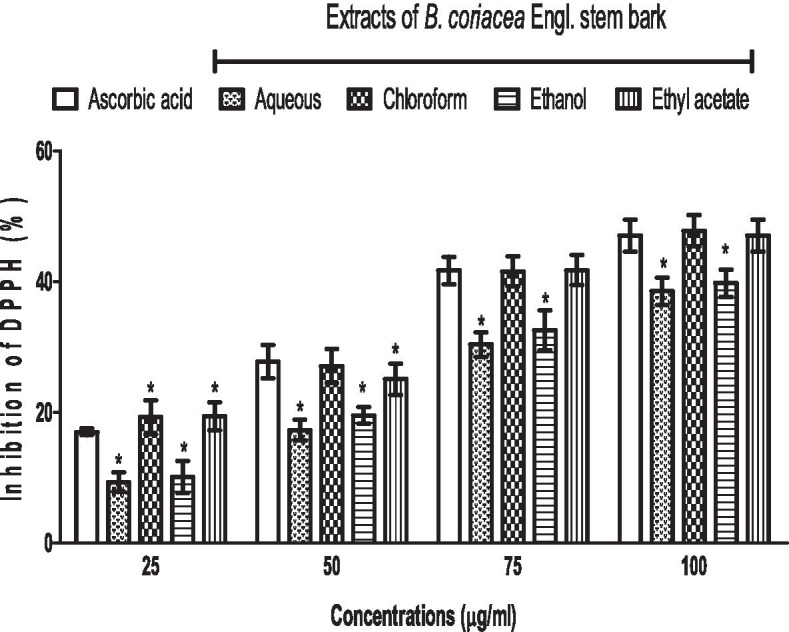
Scavenging activity of *B. coriacea* stem bark extracts on DPPH (2,2-Diphenyl-1-picrylhydrazyl) radical in vitro. *p < 0.05 vs. ascorbic acid (control)

The inhibitions of DPPH by vitamin C and BC extracts were also dose-dependent and increased as concentrations increased, reaching a peak at 100 μg/ml. Moreover, the inhibition of DPPH was increased by chloroform and ethyl acetate extracts of BC but reduced by aqueous and ethanol extracts of BC when compared to vitamin C. However, the inhibitions of DPPH caused by chloroform and ethyl acetate extracts of BC were similar across many concentrations, as the cases were for aqueous and ethanol extracts of BC (Fig. 5).

### Extracts of BC reduce ferric, albeit less than ascorbic acid in vitro

**Fig. 6:**
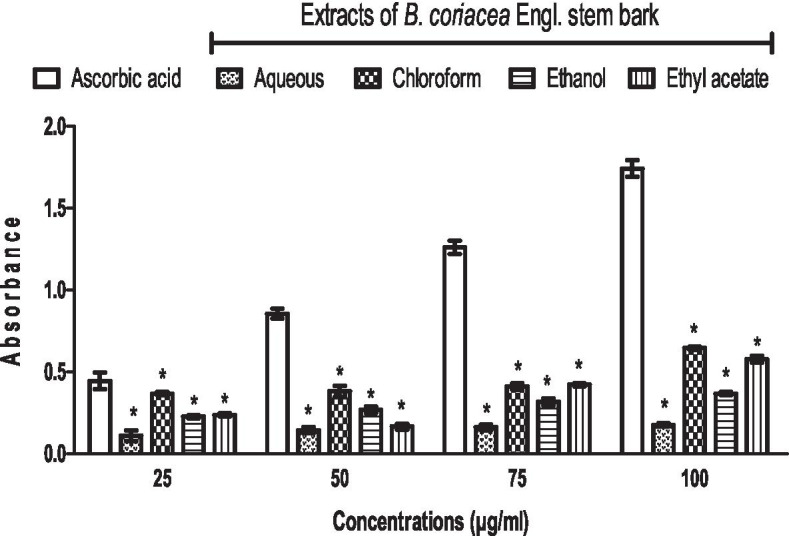
Ferric reducing the power of extracts of *B. coriacea* stem bark in vitro*.* *p < 0.05 vs. ascorbic acid (control)

Though the ferric reducing power of the extracts increased with increasing doses, they had lower reducing power compared to ascorbic acid. Moreover, the chloroform extract of BC had the highest reducing power when compared to other extracts (Fig. 6).

### Extracts of BC inhibits lipid peroxidation, albeit less than ascorbic acid in vitro

**Fig. 7:**
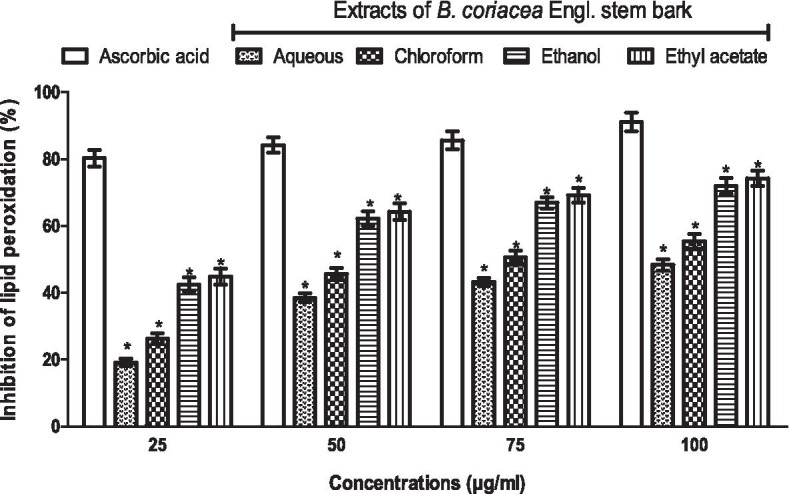
Lipid peroxidation inhibition by extracts of *B. coriacea* stem bark in vitro*.* *p < 0.05 vs. ascorbic acid (control)

The percentage inhibition of lipid peroxidation by ascorbic acid was constant across various concentrations, but higher than those of different extracts of BC. However, all the extracts of BC caused a dose-dependent increase in the inhibition of lipid peroxidation as concentrations increased, reaching a peak at 100 μg/ml. Ethyl acetate extract of BC caused the highest, while aqueous extract of BC caused the lowest inhibition of lipid peroxidation when compared to other extracts (Fig. 7).

## Discussion

*B. coriacea* Engl. (BC) is popularly called wonderful cola due to the wide ethnomedicinal use of its seed and bark for treatment of various diseases like diabetes, hypertension, catarrh, cold, infections, etc. In this study, we investigated whether it has a cytotoxic effect and explored its possible use for the treatment of cancer. Though our study validates the cytotoxic effect of 5-FU (a known anti-cancer drug), we surprisingly observed that various extracts of BC do not have a cytotoxic effect but rather increased cell viability. This BC-induced increase in cell viability was interesting to us, as it points to a likely carcinogenic effect of BC bark. Could this effect be related to inhibition of apoptotic mechanisms?

Apoptotic mechanisms are a highly complex and sophisticated energy-dependent cascade of molecular events that involve extrinsic (death receptor), intrinsic (mitochondrial) and T-cell mediated cytotoxicity (perforin-granzyme-dependent killing of the cell) pathways. Apart from the granzyme A pathway that is caspase-independent, others including extrinsic, intrinsic and granzyme B pathways initiate signals with caspases 8, 9, and 10 respectively, and later converge at the caspase-3 (execution pathway). Consequently, caspase-3 cleaves inhibitor of caspase-activated DNAse (ICAD) to release CAD, which will degrade chromosomal deoxyribonucleic acid (DNA) within the nuclei and cause DNA fragmentation, degradation of cytoskeletal and nuclear proteins, cross-linking of proteins, the formation of apoptotic bodies, expression of ligands for phagocytic cell receptors and uptake of phagocytic cells [25]. This suggests that caspase-3 is among the cross-talks of various apoptotic pathways and of physiologic importance in exploring apoptotic mechanisms. To determine whether the increase in cell viability by BC was associated with modulation of apoptosis, we measured the activity of caspase-3 in AsPC-1 treated with various concentrations of BC and also compared it with that of AsPC-1 treated with 5-FU. Our data showed that all the extracts of BC reduce caspase-3 activity, whereas 5-FU increased it. These observations mean that BC has an anti-apoptotic effect while 5-FU expectedly has a pro-apoptotic effect. It also suggests that the anti-apoptotic effect of BC is caspase-3 dependent (by inhibiting its activity).

Apart from being the cellular energy source under aerobic condition, mitochondria have been widely reported as a source of signals that initiate apoptotic cell death due to the presence of key caspase regulators in them [26]. Assembly of the apoptosome required for activating downstream caspases is induced by the release of cytochrome C from the mitochondrial intermembrane space, which is a key event in initiating apoptosis [27]. Most of the cytochrome C proteins are sequestered in the cristae, and their complete release during apoptosis requires disruption of the mitochondrial outer and inner membranes, leading to the redistribution of cytochrome C from the cristae to the intermediate space where it becomes more susceptible to release [28, 29].

The decrease in mitochondrial membrane potential (MMP) in response to many apoptotic stimuli has been well-documented [30, 31]. Opening of mitochondrial permeability transition pore (PTP) leads to swelling of the mitochondrial matrix, rupturing of the membranes and leakage of mitochondrial apoptogenic factors (e.g. cytochrome C and apoptosis-inducing factor) that lead to caspase activation. It has also been shown that PTP opening is regulated by ∆Ψm, as the probability of PTP opening increases with a decrease in ∆Ψm. There are pieces of evidence that early loss of ∆Ψm may occur independently of caspase activation. For instance, ∆Ψm loss occurred before any detectable caspase activation in resveratrol-induced apoptosis of colon tumour HCT116 cells and 2-chloro-2′-deoxyadenosine-induced apoptosis in Jurkat, JM1 and U937 cells [32, 33], and the cell death but not ∆Ψm dissipation was inhibited by z-VAD-fmk (general caspase inhibitor), suggesting that the apoptosis was independent of ∆Ψm modulation and caspase mechanism [33]. This previous observation [33] led us to further investigate if the BC-induced anti-apoptotic effect was associated with maintenance of ∆Ψm or not.

We did a ratiometric semi quantitative assessment of mitochondrial polarisation state by taking the advantage of JC-1 emission spectra shift from green to red with increasing concentration (i.e. aggregation), a method that is a more reliable indicator of ∆Ψm than other dyes [34]. In this study, incubation of AsPC-1 cells with 5-FU showed a majority of cells having the monomeric form of JC-1 dye (bright green fluorescence), which indicate de-energised mitochondria. However, fluorescence photomicrograph of cells incubated with various concentrations (20, 40 and 100 μg/ml) of different extracts of BC (aqueous, ethanol, chloroform and ethyl acetate) showed strong JC-1 aggregation (yellow), which indicates mitochondria with intact membrane potentials similar to the control cells. Moreover, extracts of BC had higher JC-1 aggregate: monomer ratio. Our observations provide strong evidence that the anti-apoptotic effect of BC is related to maintenance of intact ∆Ψm.

The previous study has suggested that inhibition of protein kinase C (PKC) decreases apoptosis by declining caspase 3 activity, inhibiting loss of ∆Ψm and protecting from loss of cell viability, suggesting that these events could share similar signalling molecule [35]. Though we did not assess PKC in this study (which is one of the study’s limitation), however, the observed increase in cell viability by BC that was accompanied with corresponding decrease in caspase 3 activity and maintenance of ∆Ψm is suggestive of the fact that BC could have similarly used PKC as a downstream signalling molecule for its anti-apoptotic effect.

Reactive oxygen species (ROS) have been demonstrated as important players in the promotion of apoptosis, DNA damage, suppression of antioxidants expression and induction of programmed cell death [36, 37]. Some neurotoxins like 6-hydroxydopamine have been reported to provoke cell apoptosis through the production of ROS and subsequent caspase 3 activation [38]. Mechanisms for ROS-induced apoptosis have been shown to include activation of caspase pathway, interference with mitochondrial protein synthesis, stimulation of the N-methyl-D-aspartate (NMDA) receptors, direct damage to the cell membrane and cell DNA [39, 40]. In vivo and in vitro studies have extensively established the protective effect of melatonin (a potent anti-oxidant) against cytotoxic activities of free radicals by enhancing anti-oxidant system and attenuating caspase 3 expression, demonstrating its anti-oxidant and anti-apoptotic potential [10, 41]. Is the anti-apoptotic effect of BC dependent on the anti-oxidant system?

Nitric oxide is important in inflammatory processes but toxic to tissues, resulting in vascular damage and other ailments at a high level. Its toxicity is heightened when it reacts with superoxide radicals to form a second reactive compound peroxynitrite anion (ONOO^−^), which forms highly reactive peroxynitrous acid (ONOOH) when protonated [42]. The DPPH is a stable free radical at room temperature that accepts an electron or hydrogen radical to become a stable diamagnetic molecule, and its assay has been widely used to determine the antioxidant capacity of plants. The present study shows that BC dose-dependently inhibits NO radical, with more inhibition as the concentration increases and peaked at 100 μg/ml. Moreover, BC causes significant inhibitions of DPPH, lipid peroxidation and ferric reduction, though not as much as ascorbic acid. Thus, the anti-apoptotic effect of BC could be partly mediated through its anti-oxidative potential by scavenging the radicals.

Are the effects of BC related to its constituents? Our qualitative analysis of BC reveals the presence of alkaloids, phenols, glycosides, terpenoids, flavonoids, saponins, tannins, and phlobatannins in aqueous and ethanol fractions. It is worthy of note that all the fractions contain phenols and flavonoids while other phytochemicals are absent in chloroform and ethyl acetate fractions of BC. The non-sugar part of saponins has a direct anti-oxidant activity that might have contributed to the high free radical scavenging capacity of BC extracts. Apart from being the largest phytochemicals, phenolics account for the largest proportion of the anti-oxidant activity of plant or its products [43] similar to the radical scavenging ability of their OH group, and a strong correlation between OH and anti-oxidant activity has been reported [44]. Flavonoids are the largest group of naturally-occurring phenolic compounds characterised by a common benzopyrone ring structure, which acts as antioxidants in various biological systems [21]. Aside anti-oxidant effect, flavonoids have been reported to elicit many other biological effects including anti-cancer, mitochondrial adhesion inhibition, antiangiogenic, antimicrobial, antiulcer, antiarthritic, and protein kinase inhibition [21]. Though it is expected that flavonoids content would be lower than phenolics content since the former is part of the latter, the reverse is the case in the present study. However, our contrary observation is consistent with previous study where *Eleutherine bulbosa* flowers had higher flavonoids (1088.33 ± 32.64 mg CE/100 g DW) than the total phenols (329.45 ± 4.55 mg GAE/100 g DW) content [21]. Our observation has been explained by some researchers that Folin-Ciocalteu’s method and Gallic acid estimate phenolic acid (a component of total phenol) rather than the total phenol content. It has also been argued that a single method can’t correctly quantify all the phonolics content in a plant sample due to the complexity of the compounds. Notwithstanding, the abundance of various phytochemicals with anti-oxidative potentials corroborates the free radical scavenging property observed with BC.

## Conclusion

**Fig. 8:**
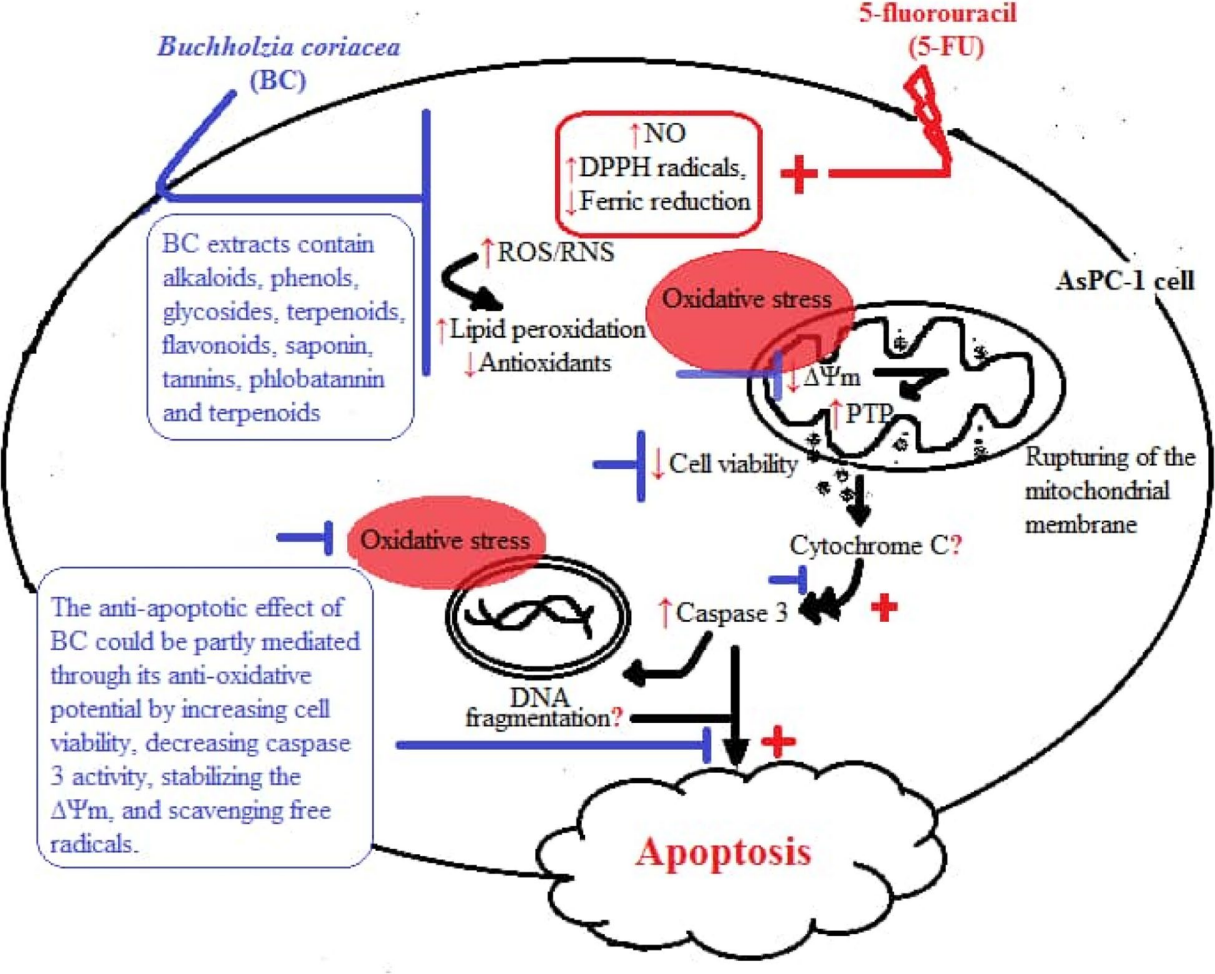
Graphical abstract

In conclusion, this study suggests that BC elicits anti-apoptotic activity in AsPC-1 in-vitro by increasing cell viability, decreasing caspase 3 activity, stabilising the ∆Ψm, and scavenging free radicals (see graphical abstract in Fig. 8). Even though BC is used ethnomedicinally as anti-cancer agent, our findings in the present study suggest that it has pro-cancer potential in-vitro, especially on pancreatic cells. Its anti-apoptotic activity in AsPC-1 could be of clinical significance, especially to counteract the effect of apoptotic agents on pancreatic cells. The study also raises a caution on the ethnomedicinal use of the BC for cancer treatment as it could exacerbate rather than ameliorate the condition. We recommend that further study should investigate the anti-apoptotic effect of BC on various other cell lines (which is part of this study’s limitation) so as to enrich our understanding on the safety of the medical use of the plant for management of cancer.

## Data Availability

The datasets used and/or analysed during the current study are available from the corresponding author on reasonable request.

## Abbreviations

AsPC-1: Human pancreatic cancer cell
5-FU: 5-fluorouracil
BC: *Buchholzia coriacea* Engl. stem bark
ΔΨm: Mitochondrial membrane potential
DPPH: 2,2-diphenyl-1-picrylhydrazyl
NO: Nitric oxide
CD4 +: Cluster of differentiation 4
AIDS: Acquired Immunodeficiency Syndrome
HL-60: Human leukaemia
HCT-8: Human colon carcinoma
MDA-MB-435: Melanoma
SF-295: Glioblastoma
ICAD: Inhibitor of caspase-activated DNAse
DNA: Deoxyribonucleic acid
MMP: Mitochondrial membrane potential
PTP: Permeability transition pore
PKC: Protein kinase C
ROS: Reactive oxygen species
NMDA: N-methyl-D-aspartate
ONOO^−^: Peroxynitrite anion
ONOOH: Peroxynitrous acid
DMSO: Dimethyl sulfoxide
DMEM: Dulbecco’s Modified Eagle Medium
PBS: Phosphate buffer saline
EDTA: Ethylenediaminetetraacetic acid
OD: Optical density
TCA: Trichloroacetic acid
NIPRD: National Institute for Pharmaceutical Research and Development
WHO: World Health Organization
SPSS: Statistical package for social sciences
ANOVA: One-way analysis of variance
